# Persistence of the midline septum of the odontoid process: anatomical and radiological study

**DOI:** 10.1007/s00276-025-03683-z

**Published:** 2025-08-04

**Authors:** Joe Iwanaga, Rarinthorn Samrid, Norio Kitagawa, Basem Ishak, Hisaaki Uchikado, Sassan Keshavarzi, Christopher M. Maulucci, Aaron S. Dumont, R. Shane Tubbs

**Affiliations:** 1 Department of Neurosurgery, Tulane Neuroscience Research Center, Tulane University School of Medicine, 131 S. Robertson St., Suite 1300, New Orleans, LA 70112 USA; 2 Department of Neurology, Tulane Neuroscience Research Center, Tulane University School of Medicine, New Orleans, LA USA; 3 Department of Structural and Cellular Biology, Tulane University School of Medicine, New Orleans, LA USA; 4 Department of Neurosurgery and Ochsner Neuroscience Institute, Ochsner Health System, New Orleans, LA USA; 5 Dental and Oral Medical Center, Kurume University School of Medicine, 67 Asahi-Machi, Kurume, Fukuoka Japan; 6 Division of Gross and Clinical Anatomy, Department of Anatomy, Kurume University School of Medicine, 67 Asahi-Machi, Kurume, Fukuoka Japan; 7 Department of Anatomy, Faculty of Medicine, Khon Kaen University, Khon Kaen, Thailand; 8 Department of Oral and Maxillofacial Anatomy, Graduate School of Medical and Dental Sciences, Institute of Science Tokyo, Tokyo, Japan; 9 Department of Neurosurgery, Heidelberg University Hospital, Heidelberg, Germany; 10 Uchikado Neuro-Spine Clinic, Fukuoka, Fukuoka Japan; 11 Department of Anatomical Sciences, St. George’s University, St. George’s, Grenada; 12 Department of Surgery, Tulane University School of Medicine, New Orleans, LA USA; 13 University of Queensland, Brisbane, Australia

**Keywords:** Cadaver, Anatomy, Dens, Odontoid process, Odontoid fracture, Axis, Cervical vertebra, Spine surgery, Computed tomography, Histology

## Abstract

**Background:**

During our screening of the computed tomography (CT) images of the craniocervical junction, we noticed that the odontoid process occasionally contains a highly ossified region in the midline consistent with a bony septum. Given its potential significance, this study aimed to investigate the highly ossified region in the midline of the odontoid process and to discuss its clinical relevance.

**Methods:**

Eleven C2 vertebrae from formalin-embalmed cadaveric heads were assigned to micro-CT examination. When the midline septum was confirmed, gross anatomical and histological observations were performed. Another 20 dry adult C2 vertebrae were randomly chosen, and the midline septum was investigated using gross anatomical and histological observations.

**Results:**

Overall prevalence of the midline septum was 22.6% (7/31) (36.7% with cadaveric C2 using micro-CT and 15% in dry C2). The midline septa were found within the anterior two-thirds in the axial view and superior two-thirds in the coronal view. The midline septum in each sample varied in its shape.

**Conclusions:**

To our knowledge, this is the first study of the persistence of the midline septum of the odontoid process. We found the overall prevalence of this septum was 22.6%. All of the midline septa were found within the anterior two-thirds in the axial view and superior two-thirds in the coronal view. Future clinical studies are necessary to further explore its potential biomechanical and surgical significance, particularly in influencing odontoid fracture patterns.

## Introduction

**Fig. 1:**
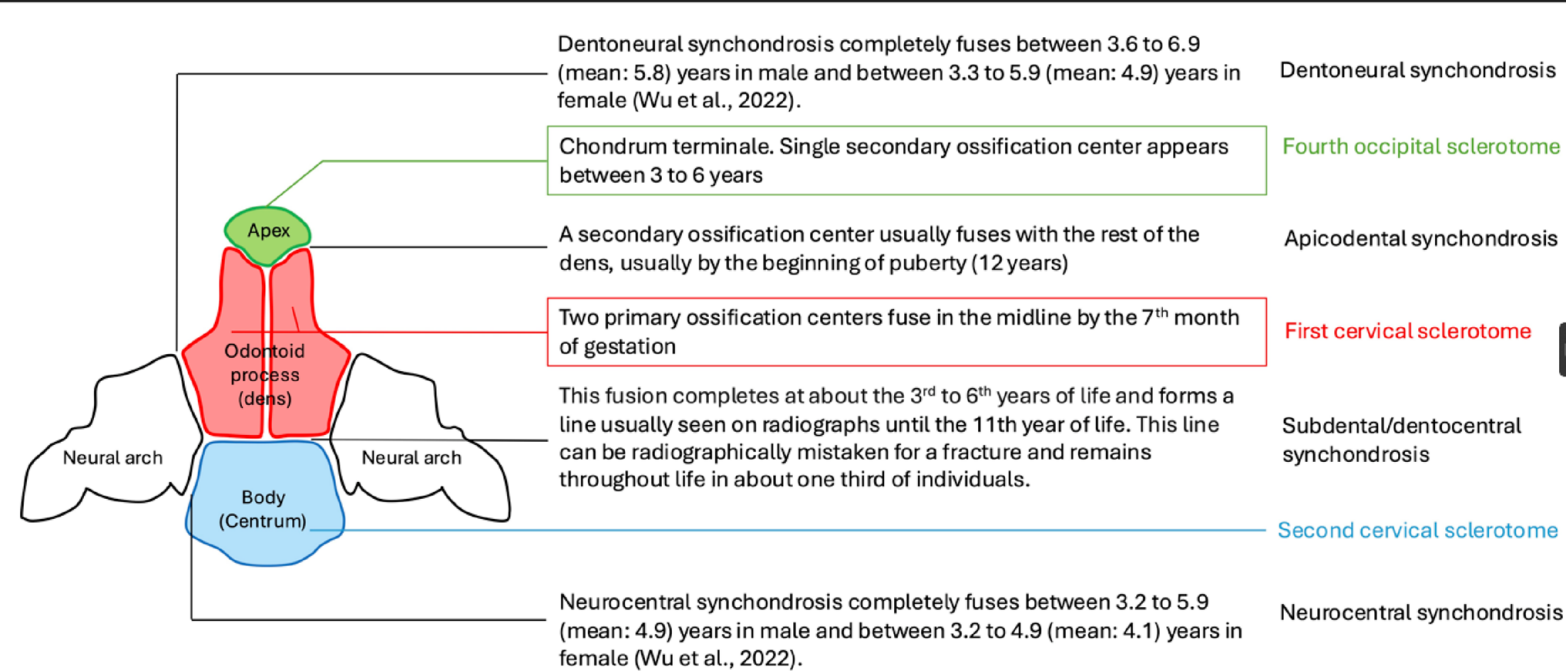
Ossification centers and synchondroses of C2

The odontoid process or dens is a projection from the C2 (axis) vertebral body and plays a crucial role at the craniocervical junction [1]. The odontoid process originates from the fourth occipital and upper two cervical sclerotomes [1]. Between the 6th and 7th weeks of gestation, the odontoid process separates from the anterior part of the atlas and migrates caudally to fuse with the body of the axis [12]. It contains primary and secondary ossification centers. The primary ossification center fuses in the midline by the 7th month of gestation (fusion between the first cervical sclerotomes) [1, 11]. Fusion of the odontoid process and body of C2 is complete between the 3rd and 6th year of life and forms a visible horizontal line usually seen on radiographs until the 11th year of life (subdental synchondrosis) [4]. Wu et al. analyzed 910 patients data to determine the ossification age of synchondroses of C1 and C2 [20] (Fig. 1). Until the 11th year, the subdental synchondrosis can be observed on radiography and is occasionally misinterpreted as an odontoid fracture [4]. Some studies have shown the persistence of the subdental synchondorosis beyond adolescence [2]. According to Gebauer et al., the remnant of the subdental synchondrosis was identified in 67% (20/30) macroscopically, and 87% (26/30) histologically in three different age groups (20–39, 40–59, and 60–80 years) [5]. Thus, the subdental synchondrosis has been well studied as it is associated with type II odontoid fractures.

**Fig. 2:**
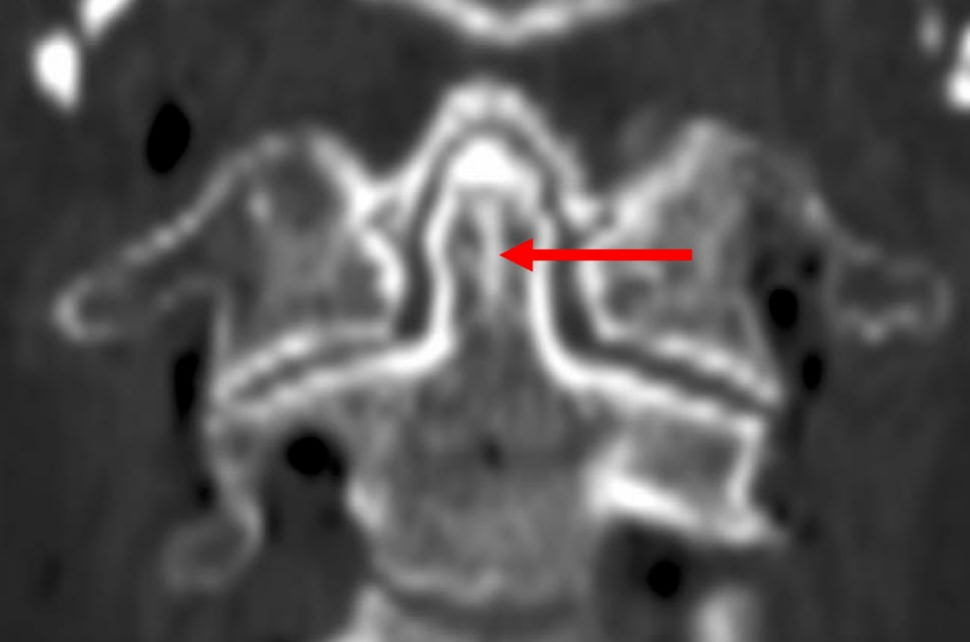
Coronal computed tomographic image. A highly ossified region in the midline of the odontoid process which looks like a bony septum (arrow)

During our computed tomography (CT) screening of the craniocervical junction, we noticed that the odontoid process occasionally has a highly ossified region in the midline, which looks like a bony septum (Fig. 2). As a midline-related variation, dens bicornis (bifid odontoid process) has been reported [1]; however, to our knowledge, there is no description of the persistence of a vertical midline septum in the odontoid process. Therefore, this study aimed to investigate the highly ossified region in the midline of the odontoid process and to discuss its clinical relevance.

## Materials and methods

### Sample selections

Eleven C2 vertebrae from formalin-embalmed cadaveric specimens (five males and six females) were imaged with micro-CT. The mean age at the time of death was 78.45 years (range 52 to 98 years) (Group 1). Another 20 dry C2 adult vertebrae were randomly chosen from our university’s collection. The age and sex were unknown (Group 2).

### Micro-CT image acquisition and observation (only for Group 1)

Images were taken using a micro-CT system with a voxel size of 0.144 mm (Quantum GX2 Micro CT, PerkinElmer, Waltham, Massachusetts, USA). The exposure volume was set at 36 mm in height and 36 mm in diameter. The CT images were taken at 90 kV and 88 µA. The axial images were transmitted in the digital imaging and communication in medicine (DICOM) format. Then, two-dimensional images of the odontoid process were reconstructed using a DICOM viewer (OsiriX) [16]. The coronal and axial sections of the micro-CT images of the odontoid process were observed. When the midline septum was observed, the superior-inferior position of the septum was recorded, i.e., the odontoid process from the base to the apex was divided into three parts: superior, middle, and inferior one-thirds.

### Gross anatomical and histological observations of cross-section images

Group 1: When the septum of the odontoid process was confirmed in the micro-CT image, the odontoid process was cut at its base using a high-speed diamond disc. The odontoid processes were decalcified using hydrochloric acid for 4 weeks and cut into coronal or axial sections.

**Fig. 3:**
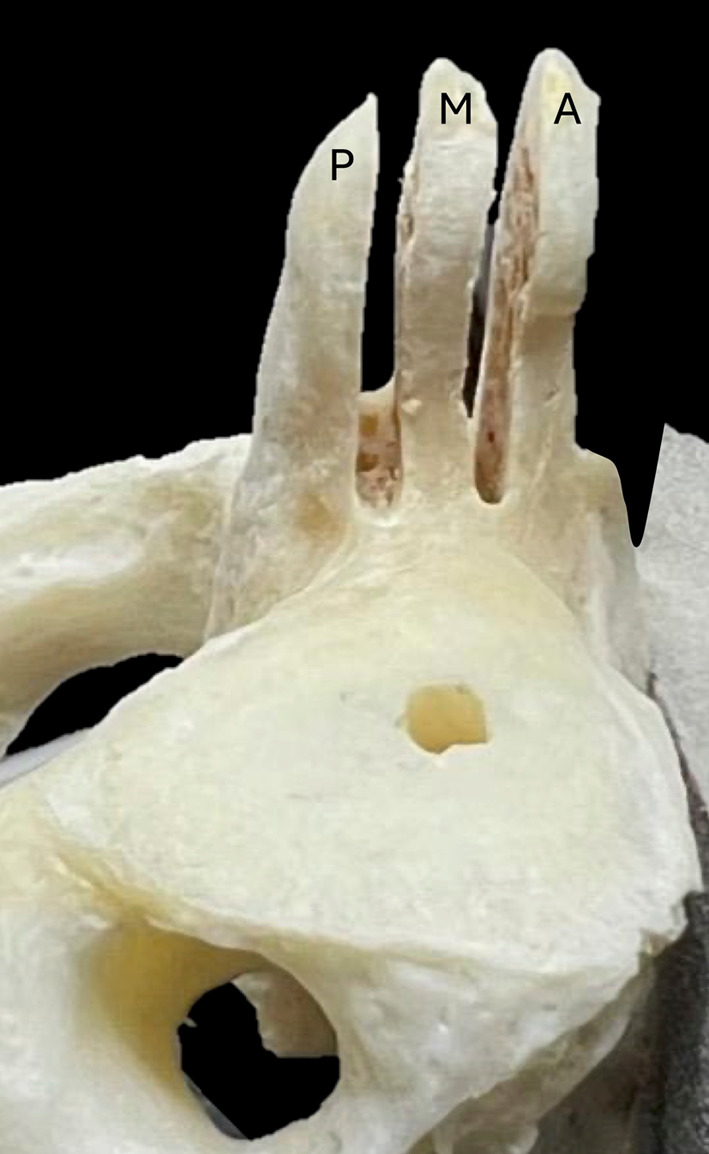
Lateral view of the C2. The odontoid process of C2 was coronally divided into three parts, i.e., anterior (A), middle (M), and posterior (P) one-thirds

Group 2: The odontoid processes were coronally cut using a high-speed drill with a diamond disc to divide them into three parts: the anterior, middle, and posterior thirds (Fig. 3). When a midline septum was observed, the odontoid process was then decalcified using hydrochloric acid.

Both groups: The tissues were embedded in paraffin. A microtome was used to cut 5 µm slices, which were then stained with Masson’s trichrome. The odontoid process was examined under a light microscope. Two anatomists (J.I. and R.S.T.) made all observations.

Statistical analysis was conducted using a student t-test to determine whether there was a significant difference in age between C2s with and without midline septum in Group 1.

The present study was performed in accordance with the requirements of the Declaration of Helsinki (64th WMA General Assembly, Fortaleza, Brazil, October 2013). The authors state that every effort was made to follow all local and international ethical guidelines and laws that pertain to the use of human cadaveric donors in anatomical research [7].

## Results

### Group 1

**Fig. 4:**
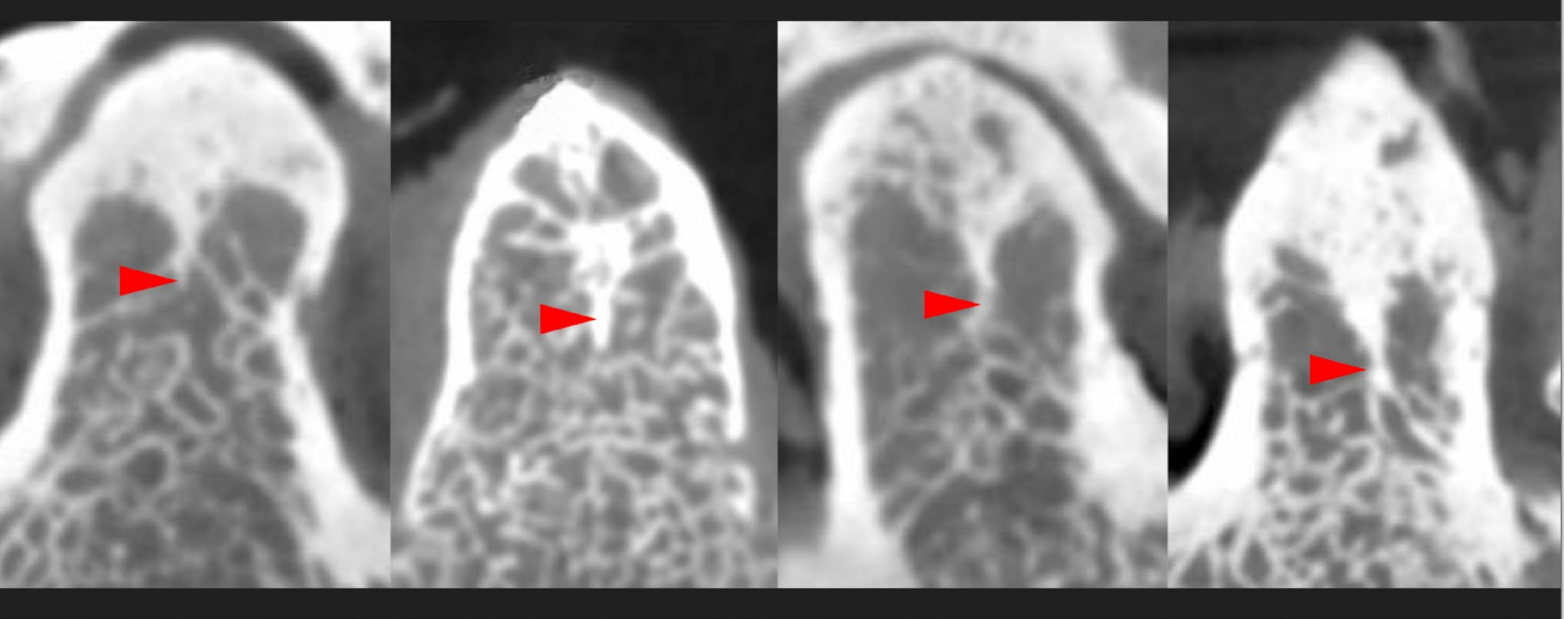
Four samples showed a highly ossified area in the midline (arrowheads) in the coronal section on micro-CT

**Fig. 5:**
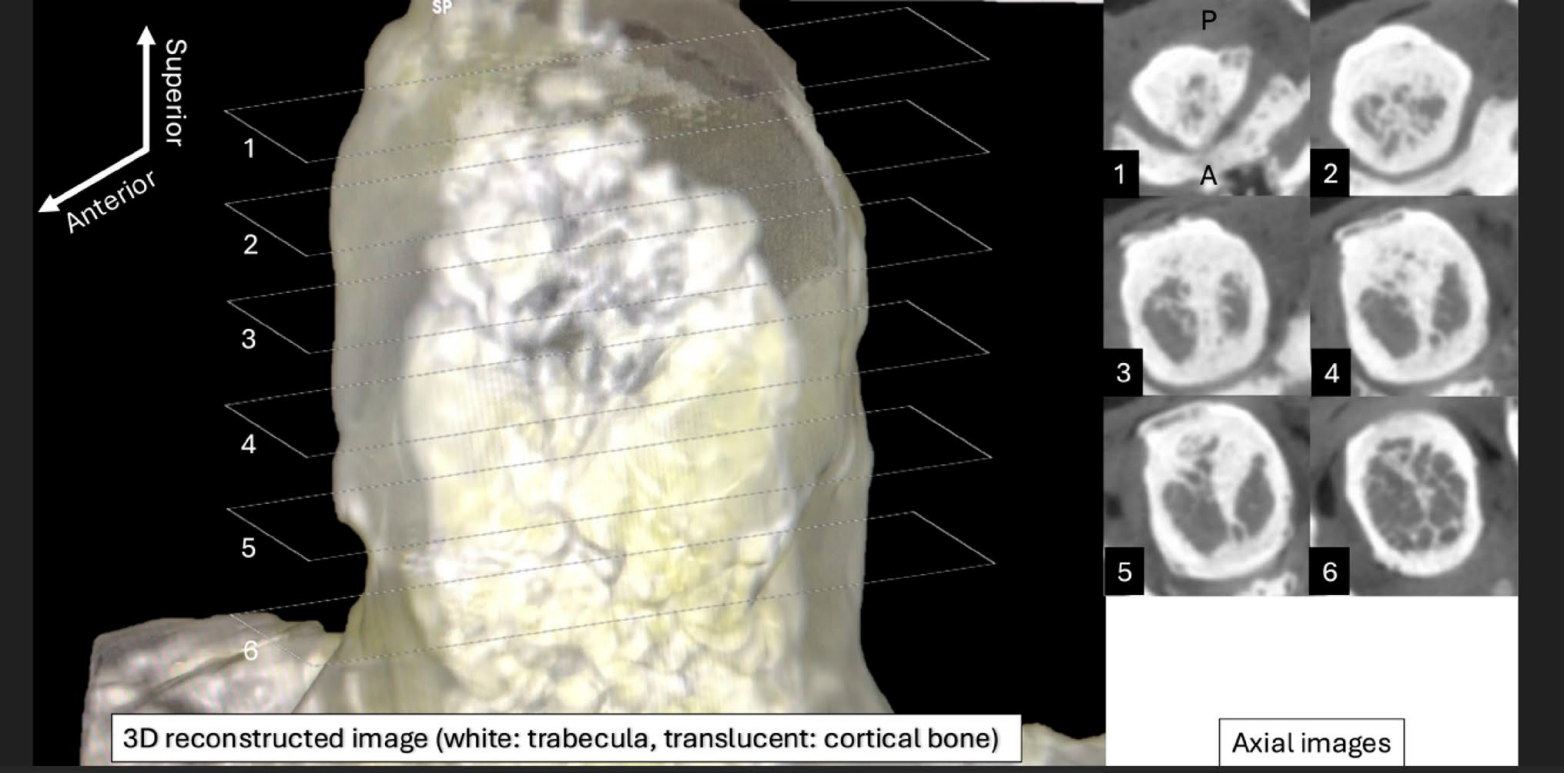
Anterolateral view of the three-dimensional reconstructed image of the odontoid process. The right axial images, labeled with numbers, correspond to the rectangle areas with the same numbers in the left image

The micro-CT images identified a highly ossified area in the midline in four out of eleven cases (36.7%) based on examining the axial and coronal section images. The midline septum was observed in the superior two-thirds (coronal view) and anterior two-thirds (axial view) of the odontoid process in all four samples (Fig. 4). As it goes posteriorly, the midline septum became wider to fuse with the posterior cortical bone (Fig. 5). There was no significant difference in age between C2 vertebrae with a midline septum and C2 vertebrae without a midline septum (77.25 years and 79.14 years, *p* = 0.84).

#### Axial section findings (n = 2)

**Fig. 6:**
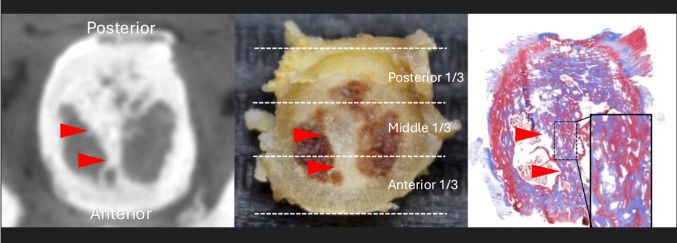
Axial sections of the midline septum (arrowheads) in micro-CT, gross anatomy, and histology (Masson’s Trichrome staining)

In two cases, the highly ossified area in the midline was observed in the anterior two-thirds of the odontoid process. The gross anatomical observation after decalcification showed that the midline bony septum was consistent with the highly ossified area identified on micro-CT images. Histological observations confirmed that the highly ossified area on micro-CT images was bone (Fig. 6). No midline septum was found in the posterior one-third.

#### Coronal section findings (n = 2)

**Fig. 7:**
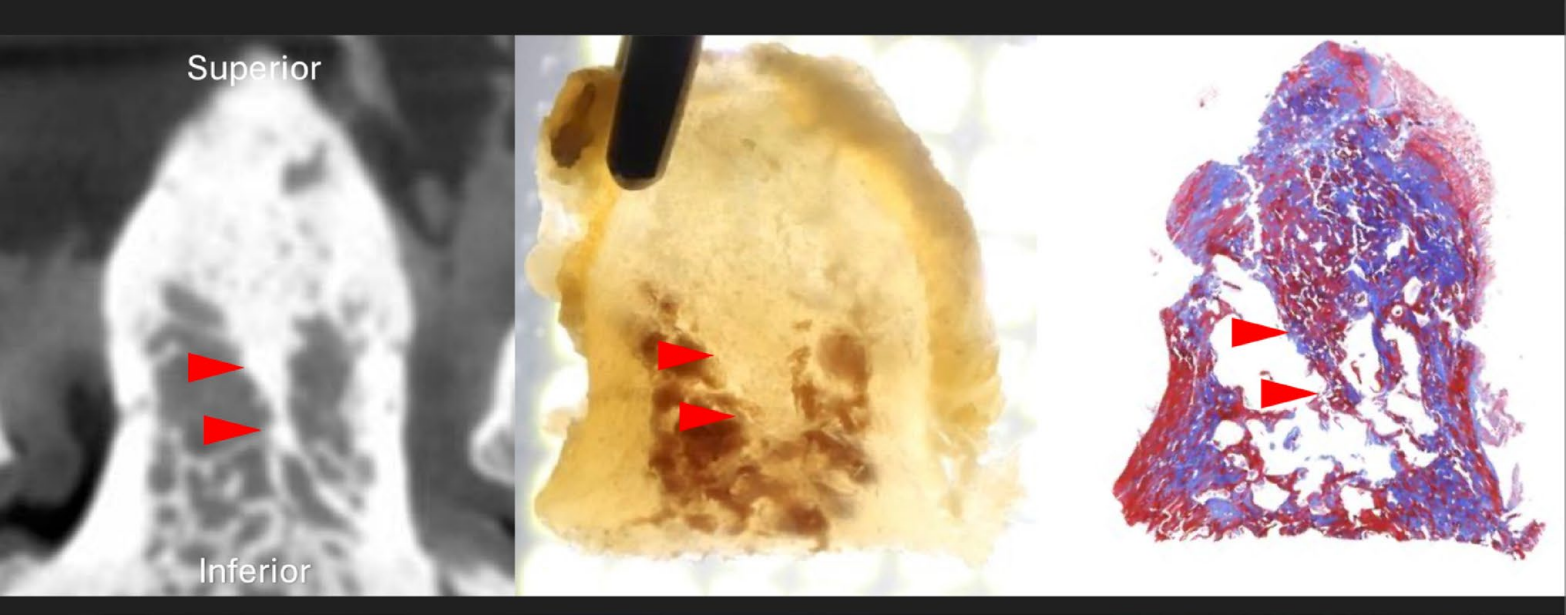
Coronal sections of the midline septum (arrowheads) on micro-CT, gross anatomy, and histology (Masson’s Trichrome staining)

In both cases, the highly ossified area in the midline was observed in the superior two-thirds of the odontoid process as a continuation of the chondrum terminale. The gross anatomical observation after decalcification showed that the midline bony septum was consistent with the highly ossified area on the micro-CT image. Histological observations confirmed that the highly ossified area seen on the micro-CT image was bone (Fig. 7). No midline septum was found in the inferior one-third.

### Group 2

#### Coronally sectioned dry C2 (n = 20) with histological observation

**Fig. 8:**
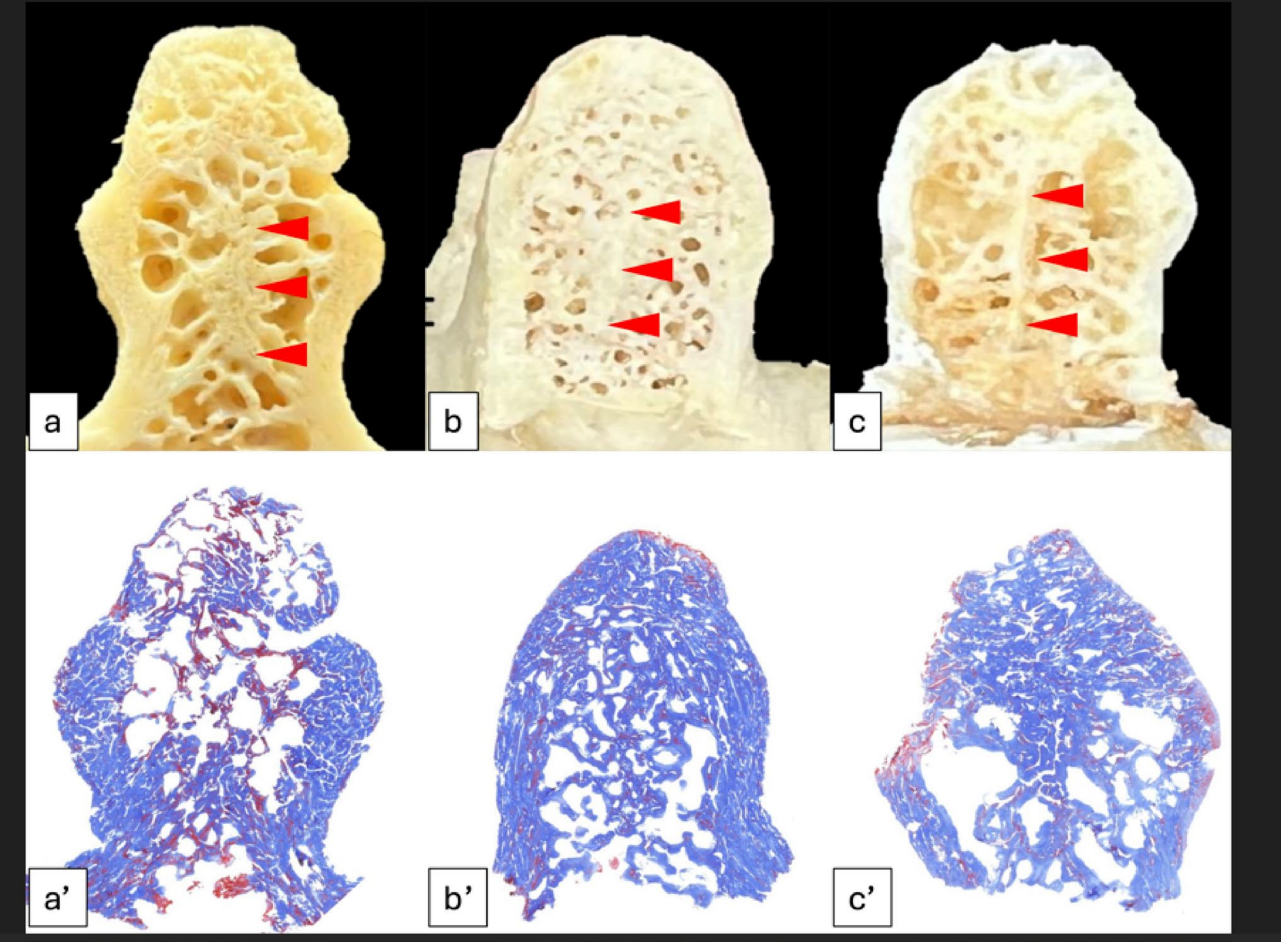
**a**–**c** Three coronally sectioned dry C2 samples with the midline septum (arrowheads), **a’**–**c’**: Histology of the midline septum in the dry C2 samples shown in figures (**a**–**c**) (Masson’s Trichrome staining)

**Fig. 9:**
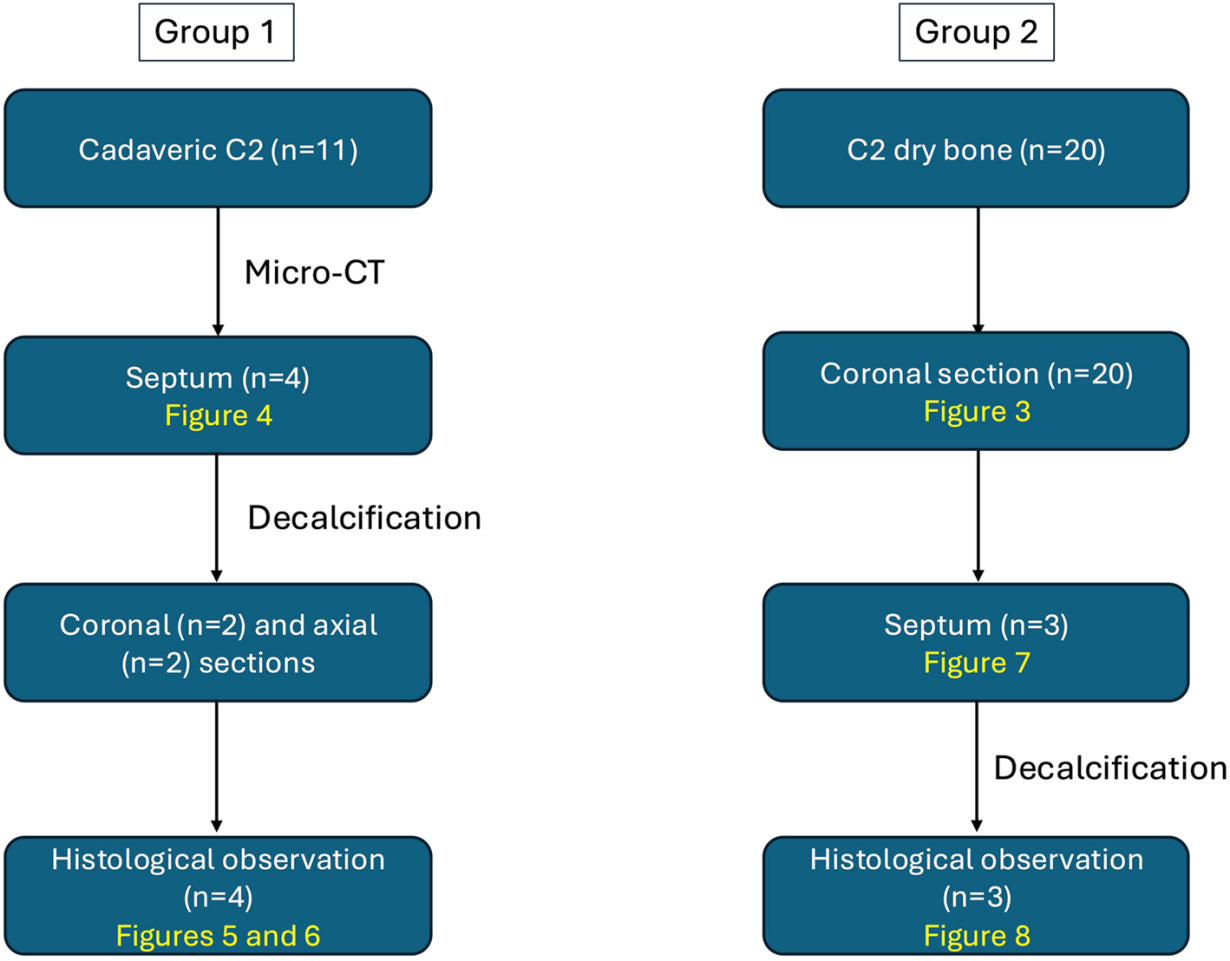
Flowcharts of the study in each group

Out of 20 dry C2 samples, the midline septum was found in three (Fig. 8a–c). In the coronal plane, the midline septum was observed in all three samples’ superior two-thirds of the odontoid process. No midline septum was found in the inferior one-third. The midline septum was observed within the anterior two-thirds (anterior one-third in two samples (10%) and anterior two-thirds in one sample (5%)). No midline septum was found in the posterior one-third. Histological observations of the three samples confirmed that the midline septum was the dense cortical bone (Fig. 8a’–c’). The overall prevalence of the midline septum was 22.6% (7/31) (36.7% with cadaveric C2 using micro-CT and 15% in dry C2). All of the midline septa were found within the anterior two-thirds in the axial view and superior two-thirds in the coronal view. The midline septum was a continuation of the chondrum terminale. The flowcharts used in this study are shown in Fig. 9.

No specimen showed signs of previous odontoid fracture or regional surgery.

## Discussion

An odontoid process is a very unique vertebra. Due to being contributed to by multiple sources during the developmental stage, it has gaps within the same bone in the early stage of life or even in later stages, i.e., apicodental, dentocentral (subdental), neurocentral, and dentoneural synchondroses [2, 3, 8]. This could result in an incorrect diagnosis, such as a fracture of the C2 due to the persistence of subdental synchondrosis in some adults. In adults, Gebauer et al. identified a remnant of the subdental synchondrosis in 67% (20/30) macroscopically and 87% (26/30) histologically [5]. Thus, anatomical and embryological knowledge directly influence the accurate diagnosis of odontoid fractures.

Odontoid fracture is reported in 10–20% of all cervical spine fractures. It is primarily found in elderly patients (65 years or older) and has a high mortality rate (37.5%) and a high risk of postoperative complications (65%) [14, 18, 19]. Odontoid fractures are classified into three types: type I, fracture at the tip of the dens; type II, fracture at the base of dens; and type III, fracture of the dens through the body of the axis [13, 14]. CT is commonly used to guide decision-making regarding the best treatment for odontoid fractures [9, 14].

The subdental synchondrosis can be misdiagnosed as a fracture as, on radiography, it is shown as a radiopaque area [5]. Even CT might fail to elucidate the subdental synchondrosis fracture, but some researchers suggested using three-dimensional reconstructed images in evaluating children as a valuable tool for diagnosis [17].

An ossification center of the chondrum terminale can be observed at 5 years old. Ossification of the odontoid process leaves a stellate fusion line in the coronal and axial planes. The apicodental synchondrosis and chondrum terminale become completely ossified at the age of 10 years. In Karwacki’s study (2012), although the midline septum was not mentioned, a fusion of the ossified chondrum terminale and apicodental synchondrosis appears very similar to the findings in the present study [10]. The midline septum might become visible when the two primary ossification centers’ fusion forms a vertical “gap” at its superior part, and excessive ossification of the apicodental synchondrosis invaginates into the gap [15].

Based on the findings of this study, the midline septum of the odontoid process is composed of dense (cortical) bone, which based on other vertebrae, is denser tissue than the surrounding trabecular bone. Therefore, with fractures the odontoid process, the midline septum of the odontoid process might affect the fracture pattern.

The presence of the midline septum as a structural feature presents intriguing possibilities regarding its biomechanical implications. Composed of dense cortical bone, the septum may enhance the strength of the odontoid process, particularly in regions subjected to high axial or rotational forces. This prompts the question of whether individuals with a midline septum are less prone to specific types of odontoid fractures or if its presence influences typical fracture patterns. Additionally, systemic conditions such as osteoporosis and osteopenia may influence the development or appearance of the septum. Future research should focus on correlating the presence of the midline septum with clinical outcomes in cases of trauma to the odontoid process or other systemic conditions. Advanced imaging modalities, such as high-resolution CT and finite element modeling, could provide deeper insights into the mechanical role of the septum in load distribution.

## Conclusions

To our knowledge, this is the first study of the persistence of the midline septum of the odontoid process. We found the overall prevalence of this septum was 22.6%. All of the midline septa were found within the anterior two-thirds in the axial view and superior two-thirds in the coronal view. We propose that this midline septum is the excessive ossification and invagination of the apicodental synchondrosis. Future clinical studies should analyze this midline septation and observe if it affects patterns of odontoid fracture.

## Data Availability

No datasets were generated or analysed during the current study.
